# Advanced lung cancer inflammation index and its predictive value for all-cause and cardiovascular mortality among osteoarthritis patients: a NHANES-based study from 2001 to 2018

**DOI:** 10.3389/fmed.2025.1593374

**Published:** 2025-05-27

**Authors:** Guiyou Wu, Dongqiang Gu, Haigang Jia, Lei Hong, Xishun Wang

**Affiliations:** ^1^Senior Department of Orthopedics, The Fourth Medical Center of Chinese PLA General Hospital, Beijing, China; ^2^National Clinical Research Center for Orthopedics, Sports Medicine and Rehabilitation, Beijing, China; ^3^Department of Orthopedics, Capital Center for Children’s Health, Capital Medical University, Beijing, China

**Keywords:** osteoarthritis, advanced lung cancer inflammation index, all-cause mortality, cardiovascular mortality, NHANES

## Abstract

**Background:**

Globally, the occurrence of osteoarthritis (OA) is increasing, and the intensity of the condition is frequently determined by the level of inflammation and nutritional status in a person. The Advanced Lung Cancer Inflammation Index (ALI), a pioneering metric, merges two pivotal elements. For instance, it integrates markers of inflammation with measures of nutritional status. This research seeks to delineate the link between ALI and the risk of mortality, whether from universal causes or cardiovascular disease (CVD), particularly in the OA-affected population.

**Methods:**

This research drew upon the National Health and Nutrition Examination Survey (NHANES) database from 2001 to 2018. The study aimed to thoroughly examine the potential association between the ALI and the risk of all – cause as well as CVD – related mortality in patients with OA. The analysis employed weighted Kaplan–Meier methods to estimate survival probabilities and multivariate-adjusted Cox regression models to quantify hazard ratios, adjusting for confounding variables. To assess potential nonlinear associations between ALI and mortality outcomes, a restricted cubic spline (RCS) approach was implemented. Additionally, stratified analyses (e.g., by age, sex, or comorbidities) and interaction tests were conducted to evaluate effect modification and validate the robustness of findings across subgroups. These methodological strategies ensured comprehensive exploration of the ALI-mortality link in OA patients while minimizing bias and enhancing generalizability.

**Results:**

This research delved into the health records of 2,133 individuals, revealing a notable connection: those with OA and higher ALI scores exhibited significantly lower rates of both overall and CVD-related fatalities. Specifically, a non-linear relationship characterized by an L-shaped curve was observed, indicating that ALI scores below a critical threshold (inflection point at 61.18) were associated with progressively elevated mortality risks, while scores above this threshold exhibited a protective effect against mortality. This pattern was consistent for both all-cause and CVD mortality outcomes, underscoring a threshold-dependent modulation of risk by ALI in OA patients. A subgroup analysis showed a stronger link between ALI scores and all-cause mortality in female OA patients, while no significant interaction was found concerning CVD mortality. Furthermore, time-dependent Receiver Operating Characteristic (time-ROC) curve analysis emphasized the strong predictive ability of the ALI.

**Conclusion:**

This cohort study shows that ALI is effective in forecasting mortality outcomes for OA patients, highlighting its clinical significance for risk assessment and prognosis in this population.

## Introduction

Osteoarthritis (OA) is the most prevalent form of arthritis, impacting around 250 million individuals globally and causing significant disability (1). This condition is characterized by the gradual development of its pathophysiological aspects, Notably, the structure and mechanical behavior of the articular cartilage and subchondral bone undergo substantial transformations, alongside a relatively mild presence of synovitis. These modifications lead to the emergence of pain, stiffness, and a progressive reduction in joint mobility, which significantly diminishes the quality of life for those affected (2). Extensive research has demonstrated that OA extends its impact beyond the skeletal system, exhibiting a significant association with elevated rates of both all-cause mortality and cardiovascular disease (CVD) mortality (3). Based on a thorough review of multiple studies, it has been found that people suffering from OA are at a 13% higher risk of dying from any cause and have a 21% greater chance of dying due to CVD compared to the general public (4). These findings underscore the critical need for the development of reliable predictors to accurately assess mortality risk in OA patients.

In the pathophysiology of OA, chronic low-grade inflammation is a key player. Of particular significance are several protein biomarkers, namely interleukin-1 (IL-1), tumor necrosis factor (TNF-*α*), and matrix metalloproteinases (MMPs), have been identified as indicative of inflammation, cartilage degradation, synovitis, and pain characteristic of OA (5–7). This inflammatory response not only speeds up the progression of OA but also connects the condition to other health issues, including CVD (8). Additionally, inflammation worsens sarcopenia in individuals with OA, subsequently elevating the risk of mortality (9). Although clinical inflammatory indicators like C-reactive protein (CRP) and erythrocyte sedimentation rate (ESR) are frequently utilized in the evaluation of OA, they have shown limited effectiveness in predicting disease outcomes (10). In response to this challenge, investigators have been investigating alternative inflammatory biomarkers that better capture the complex relationship between inflammation, nutritional status, and the outcomes associated with OA.

The newly introduced Advanced Lung Cancer Inflammation Index (ALI) is designed to assess the cumulative impact of both nutritional and inflammatory components in chronic diseases. The ALI is derived from a combination of specific metrics, including body mass index (BMI), serum albumin concentrations, and the neutrophil-to-lymphocyte ratio (NLR) (11). Notably, reduced ALI scores, which signify malnutrition and systemic inflammatory states, have been correlated with adverse survival outcomes in diverse pathologies such as cancer, chronic kidney disease, and coronary artery disease (12–15).

Recent studies have underscored the pivotal role of systemic inflammation and nutritional status in the progression and prognosis of OA. For instance, Guo et al. analyzed data from the National Health and Nutrition Examination Survey (NHANES) and found that lower ALI levels were significantly associated with increased risks of all-cause and cardiovascular mortality in OA patients, exhibiting an L-shaped non-linear relationship (16). Similarly, Zhou et al. demonstrated that elevated systemic immune–inflammation index (SII) and systemic inflammation response index (SIRI) were linked to higher all-cause and cardiovascular mortality among individuals with OA (17). Furthermore, a study published in BMC Musculoskeletal Disorders reported that a higher Dietary Inflammatory Index (DII), reflecting a pro-inflammatory diet, was associated with increased all-cause mortality in OA patients (18). These findings collectively highlight the significance of integrating inflammatory and nutritional assessments in evaluating mortality risk among OA patients. Given that ALI encompasses both inflammatory and nutritional components, its application as a prognostic marker in OA is well-founded.

While inflammation and heightened mortality rates are key indicators, the precise impact of ALI on OA is still uncertain. This study aims to examine the link between the ALI and mortality rates, specifically overall and cardiovascular deaths, in OA patients. We leveraged data from the NHANES, spanning 2001 to 2018, for this analysis.

The NHANES dataset, characterized by its comprehensive cross-sectional analysis of a diverse and nationally representative population, is instrumental in our assessment of ALI and the examination of long-term mortality patterns (19). Based on this premise, we propose the hypothesis that among OA patients, lower ALI values will correlate with increased all-cause and CVD mortality in comparison to those with higher ALI values.

## Materials and methods

Our research is designed as a forward-looking study, tracking a group of people over time to observe health outcomes. We utilized data from the NHANES, a comprehensive survey conducted biennially since 1999. NHANES is principally aimed at conducting intermittent appraisals of the health and dietary habits of Americans. To uphold the ethical benchmarks and the sanctity of the research, every protocol associated with NHANES underwent scrutiny and received the green light from the ethics panel at the National Center for Health Statistics. Moreover, informed consent was obtained in writing from each participant, signifying their agreement and voluntary engagement in the survey (20).

### Study population

To determine the presence of OA among participants, we relied on their self-reported medical histories. These histories were collected through detailed, face-to-face interviews at participants’ homes, conducted by trained interviewers using a computer-assisted method known as CAPI. The key part of this process was the Medical Conditions Questionnaire (MCQ), which included specific questions about their health. One of the crucial questions asked during the family interview segment was, “Has a doctor or any healthcare professional ever told you or someone in your family that they have arthritis?”

Respondents had the option to reply with “yes” or “no.” For the purpose of distinguishing the types of arthritis, those who responded affirmatively were further questioned about the specific type of arthritis they were diagnosed with. Responses indicating “Osteoarthritis or degenerative arthritis” were classified as OA patients. Those who reported “Chronic inflammatory arthritis,” “Arthritis associated with psoriasis,” “Additional,” “Unclear,” or “Declined” were excluded from the study. From the total of 91,351 participants surveyed between 2001 and 2018, the following were excluded: (a) those under 20 years old (*n* = 50,201); (b) individuals with incomplete mortality data (*n* = 47,876); (c)participants without arthritis (*n* = 12,829); (d) subjects not diagnosed with OA (*n* = 4,676); (e) cases with missing data on nutritional-inflammatory markers (*n* = 385); and (f) entries with any missing covariate data (*n* = 2,158). The final analysis included a sample of 2,133 OA patients who met all eligibility criteria.

### Assessment of ALI

To calculate the ALI, we used a specific formula: ALI = BMI × Alb/NLR, Here’s a breakdown: BMI is computed by dividing an individual’s mass in kilograms by the square of their stature in meters. The concentration of serum albumin (Alb) is quantified in grams per deciliter, serving as an indicator of albumin levels in the bloodstream. The Neutrophil-to-Lymphocyte Ratio (NLR) is derived by dividing the aggregate count of neutrophils by the corresponding count of lymphocytes in the blood (21). Patients were categorized into four groups based on the quartiles of ALI: Q1 (ALI ≤ 43.29), Q2 (43.29 < ALI ≤ 61.32), Q3 (61.32 < ALI ≤ 82.58), and Q4 (ALI > 82.58). Higher ALI scores imply better nutritional status and lower inflammation levels among the subjects. Additional computations for other nutritional and inflammatory markers are presented in Supplementary Table S1.

### Evaluation of mortality

Information from the National Center for Health Statistics was employed to establish all-cause and cardiovascular mortality rates, with the end of follow-up defined as December 31, 2019. Causes of death were determined based on data from the National Death Index (NDI), which provides cause-specific mortality exclusively coded using the International Classification of Diseases, 10th Edition (ICD-10), regardless of the year of death. Cardiovascular-related deaths were identified using ICD-10 codes ranging from I00 to I078 (22). The exclusive use of ICD-10 ensures consistency across all NHANES survey cycles and improves the specificity of cardiovascular outcome classification compared to older coding systems such as ICD-9-CM.

### Covariate definitions

To investigate the effects of confounding variables on the research outcomes., several covariates were incorporated, including age, gender, ethnicity, education level, alcohol use, tobacco use, BMI, waist circumference, income-poverty ratio (PIR), and the presence of diabetes mellitus (DM), hypertension, hyperlipidemia, CVD, cancer. What’s more, the levels of alanine aminotransferase (ALT) and aspartate aminotransferase (AST) were considered. The choice of these covariates was informed by an extensive review of current literature. Detailed categorizations of the variables and their respective units are presented in Table 1.

**Table 1:** Baseline characteristics of the study population stratified according to the advanced lung cancer inflammation index.

Characteristics	Advanced lung cancer inflammation index				*p*-value
	Q1 (*N* = 534)	Q2 (*N* = 533)	Q3 (*N* = 533)	Q4 (*N* = 533)	
Age (years)	65.84 ± 14.14	61.24 ± 14.03	60.66 ± 12.96	59.09 ± 12.52	<0.001
Male	232 (43.4%)	235 (44.1%)	202 (37.9%)	172 (32.3%)	0.039
Race, *n* (%)					<0.001
Mexican American	32 (6.0%)	47 (8.8%)	48 (9.0%)	36 (6.8%)	
Non-Hispanic Black	27 (5.1%)	49 (9.2%)	60 (11.3%)	144 (27.0%)	
Non-Hispanic White	423 (79.2%)	384 (72.0%)	374 (70.2%)	275 (51.6%)	
Others	52 (9.7%)	53 (9.9%)	51 (9.6%)	78 (14.6%)	
Education level, *n* (%)					0.443
< High school	77 (14.4%)	81 (15.2%)	74 (13.9%)	74 (13.9%)	
> High school	351 (65.7%)	324 (60.8%)	348 (65.3%)	338 (63.4%)	
High school	106 (19.9%)	128 (24.0%)	111 (20.8%)	121 (22.7%)	
Drinking alcohol, *n* (%)					0.423
Heavy drinker	82 (15.4%)	67 (12.6%)	79 (14.8%)	62 (11.6%)	
Low to moderate drinker	401 (75.1%)	414 (77.7%)	393 (73.7%)	407 (76.4%)	
Non-drinker	51 (9.6%)	52 (9.8%)	61 (11.4%)	64 (12.0%)	
Smoke, *n* (%)					0.015
Current	90 (16.9%)	104 (19.5%)	67 (12.6%)	99 (18.6%)	
Former	223 (41.8%)	205 (38.5%)	213 (40.0%)	166 (31.1%)	
Never	221 (41.4%)	224 (42.0%)	253 (47.5%)	268 (50.3%)	
BMI (kg/m^2^), *n* (%)					<0.001
<25	216 (40.4%)	127 (23.8%)	69 (12.9%)	52 (9.8%)	
≥30	115 (21.5%)	221 (41.5%)	289 (54.2%)	343 (64.4%)	
25 to < 30	203 (38.0%)	185 (34.7%)	175 (32.8%)	138 (25.9%)	
Waist circumference (cm)	97.07 ± 14.78	102.89 ± 15.31	106.17 ± 15.73	109.03 ± 16.81	<0.001
PIR, *n* (%)					0.098
<1.3	88 (16.5%)	96 (18.0%)	100 (18.8%)	134 (25.1%)	
≥3.5	229 (42.9%)	225 (42.2%)	251 (47.1%)	239 (44.8%)	
1.3 to <3.5	217 (40.6%)	212 (39.8%)	182 (34.1%)	160 (30.0%)	
Diabetes mellitus, *n* (%)	318 (59.6%)	308 (57.8%)	302 (56.7%)	334 (62.7%)	0.421
Hypertension, *n* (%)	377 (70.6%)	376 (70.5%)	387 (72.6%)	408 (76.5%)	0.024
Hyperlipidaemia, *n* (%)	486 (91.0)	478 (89.7)	492 (92.3)	490 (91.9)	0.604
CVD, *n* (%)	114 (21.3%)	95 (17.8%)	76 (14.3%)	74 (13.9%)	0.33
Cancer, *n* (%)	137 (25.7%)	103 (19.3%)	101 (18.9%)	92 (17.3%)	0.498
AST (U/L)	25.10 ± 14.89	24.99 ± 11.96	25.79 ± 13.15	26.47 ± 13.43	0.651
ALT (U/L)	22.60 ± 16.07	23.61 ± 13.69	24.71 ± 16.10	25.44 ± 14.07	0.001

Mean ± standard deviation for continuous variables, the *p*-value was calculated by the weighted linear regression model; *n* (%) for categorical variables, the *p*-value was calculated by the weighted chi-square test. ALT, Alanine aminotransferase; AST, Aspartate aminotransferase.

In our examination, participants were classified into four ethnic categories: Mexican Americans, Non-Hispanic Blacks, Non-Hispanic Whites, and a miscellaneous group encompassing other ethnicities. For educational achievement, we identified three levels: those who had not attained a high school diploma, individuals who had completed high school, and those who had pursued education beyond the high school level. Smoking habits were differentiated into three classifications: individuals who currently smoke, those who previously smoked but have since quit, and individuals who have never smoked. Alcohol consumption was divided into three patterns: heavy drinkers, moderate to low drinkers, and abstainers (23). We also classified BMI into three categories to reflect weight status: normal weight (below 25 kg/m^2^), overweight (25 to under 30 kg/m^2^), and obese (30 kg/m^2^ and above). Additionally, we stratified the PIR into three ranges to assess economic status: below 1.3, from 1.3 to below 3.5, and 3.5 or higher.

In our assessment of diabetes status, we relied on multiple indicators. Firstly, participants who self-reported a prior diagnosis of diabetes were considered diabetic. Secondly, individuals currently using insulin or antidiabetic medications were also classified as having diabetes. Additionally, we employed specific biochemical criteria for diagnosis: To diagnose diabetes, the criteria included a fasting plasma glucose concentration of 7.0 mmol/L or higher, an oral glucose tolerance test (OGTT) outcome surpassing 11.1 mmol/L, a random plasma glucose level exceeding 11.1 mmol/L, or a glycated hemoglobin A1c level at or above 6.5%. These combined methods ensured a comprehensive and accurate identification of diabetes cases. Hypertension was identified through self-reports of being diagnosed with high blood pressure, having received this information on two separate occasions, or currently taking prescription medication for high blood pressure. The NHANES guidelines categorize hypertension as having an average blood pressure reading of at least 130 mmHg systolic or 80 mmHg diastolic, following a minimum of three readings. The presence of hyperlipidaemia was ascertained through self-reported medical diagnoses, the intake of lipid-lowering drugs as prescribed, or by fulfilling any one of the following benchmarks: In our investigation, we identified elevated total cholesterol levels exceeding 200 mg/dL, triglyceride concentrations surpassing 150 mg/dL, and high-density lipoprotein (HDL) cholesterol levels in males beyond 130 mg/dL. The occurrence of CVD among participants was determined based on their self-reported medical histories, which included significant conditions such as stroke, angina, myocardial infarction, coronary heart disease, and heart failure. For cancer, a diagnosis was validated by a positive self-report, an age of first diagnosis spanning from 1 to 80 years, or by specifying the particular type of cancer encountered. For further details on all covariates used in our analysis, please refer to the comprehensive data available at: www.cdc.gov/nchs/nhanes/index.htm.

### Statistical analysis

To ensure the integrity of our analysis in line with the sophisticated NHANES sampling methodology, we incorporated sample weighting, clustering, and stratification into our analytical process. The initial distribution of the ALI was presented in quartiles (Q1-Q4), providing a clear visual representation of the data. In our analysis, continuous variables were summarized using weighted means, accompanied by standard errors (SE) to indicate precision. For categorical data, we presented unweighted frequencies to show the raw counts, and to provide a more complete picture, we also included weighted percentages.

In the course of our data analysis, we applied weighted linear regression to handle continuous outcomes and relied on chi-square tests for categorical variables, using sturdy methods to carefully examine the dataset. To gauge the predictive power of the ALI in terms of mortality, we performed a time-ROC analysis, benchmarking it against a range of nutritional and inflammatory indicators from blood tests to achieve a thorough evaluation. Our quest to understand the connection between ALI levels and mortality hazards led us to use the Kaplan–Meier estimator for a detailed exploration. By employing this approach, we were able to investigate the relationship between ALI levels and the hazards of both overall and CVD mortality in the OA patient population, providing clarity on the potential effects of ALI on patient outcomes.

Commencing our investigation, we sought to identify the determinants of all-cause and CVD mortality among patients with OA. Our analytical journey began with a univariate Cox regression, providing a preliminary glimpse into the factors at play. To further unravel the intricate relationship between the ALI and mortality risks, we then deployed a multivariate Cox proportional hazards regression model, meticulously adjusting for a constellation of covariates. Our study framework comprised three distinct analytical models: a baseline unadjusted model (model 1), a model fine-tuned for demographic and socioeconomic variables (including age, sex, ethnicity, PIR, and education) (model 2), and a comprehensive model incorporating all relevant covariates (model 3). Each model was meticulously crafted to assess the ALI-OA relationship through hazard ratio calculations.

In our investigation into the potential non-linear correlation between ALI levels and mortality rates among individuals with OA, we applied Restricted Cubic Spline (RCS) analysis. Upon observing a non-linear trend, we initiated a recursive algorithm to precisely locate the inflection point. In our pursuit to enhance the comprehension of the connection between ALI and OA, we performed thorough subgroup analyses, taking into account essential variables such as age, gender, ethnicity, BMI, diabetes, hypertension, CVD, and cancer. Statistical analyses were conducted using R software, version 4.4.0, and a two-sided *p*-value of less than 0.05 was set as the benchmark for statistical significance. This rigorous analytical approach ensured a comprehensive examination of the ALI-OA relationship and its implications for mortality risks in OA patients.

## Results

### Baseline characteristics of the study participants

**Figure 1:**
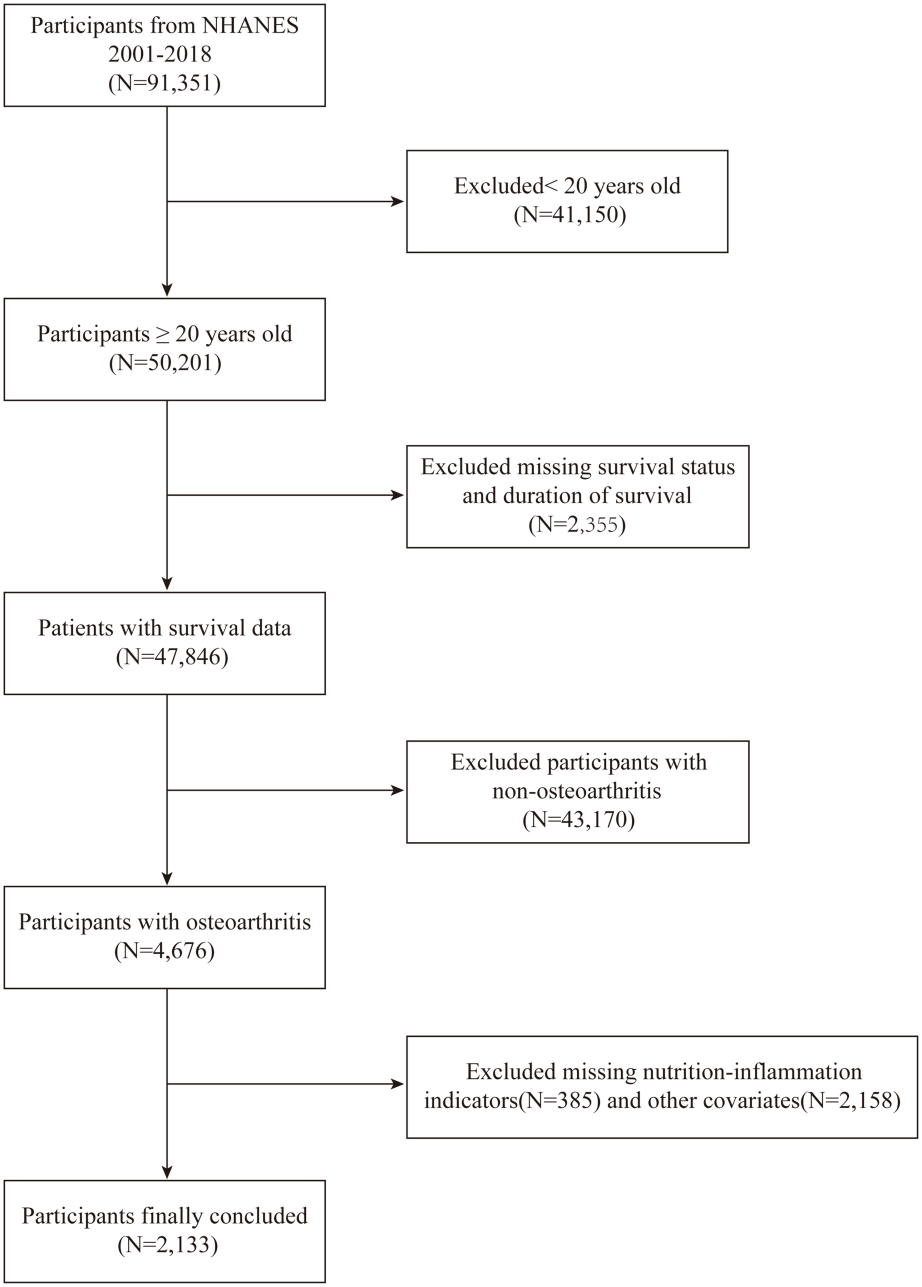
Flowchart for selecting the study population.

Our study began with the identification of 91,351 individuals from the NHANES cohorts, spanning a period from 2001 to 2018. Following a rigorous application of defined inclusion and exclusion criteria, the study population was narrowed down to 2,133 eligible participants. The flow of participants through the study is visually represented in Figure 1. Participants were then stratified into four distinct groups based on their ALI levels, with the initial group numbering 534 individuals, and the subsequent three groups each comprising 533 individuals. This categorization facilitated a balanced comparative analysis across the groups. Additionally, the demographic and clinical characteristics of these groups were further examined to ensure homogeneity in the study population.

In comparison to the 4 Quantile groups exhibited notable differences, these groups were younger, had a higher percentage of female participants, and a lower proportion of White individuals, whereas the percentage of Black individuals increased. Additionally, these groups demonstrated a higher prevalence of non-smokers, elevated BMI values, and increased waist circumference. The proportion of individuals with hypertension also rose, alongside elevated levels of AST. However, similar levels of education, alcohol consumption, PIR, diabetes mellitus, hyperlipidemia, CVD, cancer, and AST were observed across all groups. Detailed information is provided in Table 1.

### Screening the best nutrition-inflammation indicators

**Figure 2:**
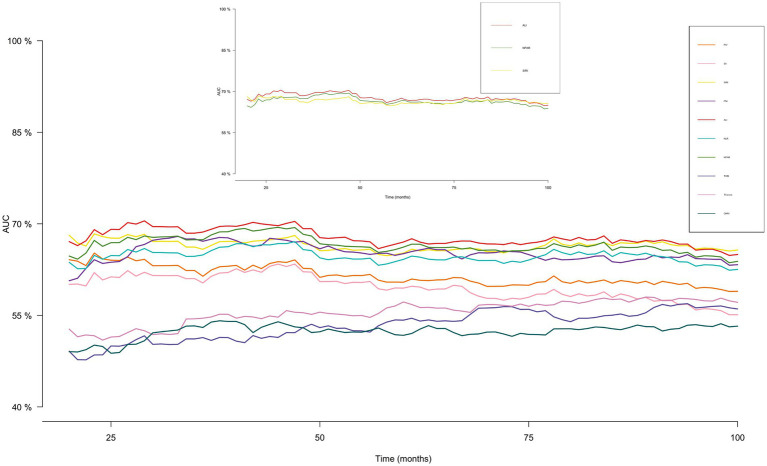
Time-dependent ROC of inflammation and nutrition-related indicators for diagnosing overall survival in OA patients.

The effectiveness of ten nutritional and inflammatory indicators in predicting overall survival among patients with OA was evaluated using time-ROC analysis. The AUC value for the ALI was found to be superior to that of more than ten other nutritional or inflammatory indicators (Figure 2). The methods used to calculate the various nutritional and inflammatory indicators are detailed in Supplementary Table S1.

### Kaplan–Meier analysis

**Figure 3:**
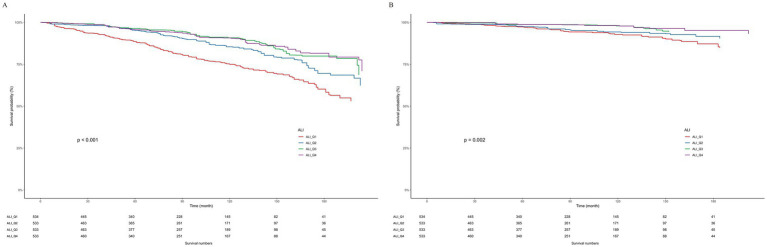
Weighted Kaplan–Meier curves for the association of ALI with **(A)** all-cause and **(B)** CVD mortality in OA patients.

In an effort to elucidate the connection between all-cause and CVD mortality in patients afflicted with both ALI and OA, we utilized the Kaplan–Meier analysis method. Within our cohort of 2,133 OA patients, we documented 371 fatalities due to all causes, among which 106 were specifically linked to CVD. Figure 3 presents the Kaplan–Meier survival curves, effectively illustrating the differential mortality rates associated with varying ALI levels among OA patients, specifically for all-cause and CVD-related deaths. Our investigation uncovers a notable inverse association: elevated ALI levels exhibit a reverse correlation with the hazards of both overall and CVD-related mortality, as evidenced by *p*-values of < 0.001 and 0.002, respectively.

### Association of ALI with all-cause and CVD mortality among OA

Table 2 delineates the outcomes of three Cox regression models, with Model 3 standing out due to its inclusion of a more exhaustive array of covariates. This comprehensive approach in Model 3 effectively mitigates the influence of potential confounders, thereby elevating the significance of its findings and making it the cornerstone of our study’s analysis.

**Table 2:** Association of advanced lung cancer inflammation index (ALI) with all-cause and CVD mortality among OA patients.

Subgroup	Model 1^a^		Model 2^b^		Model 3^c^	
	HR (95% CI)	*p*-value	HR (95% CI)	*p*-value	HR (95% CI)	*p*-value
All-cause mortality						
ln ALI	0.38 (0.28–0.51)	<0.001	0.54 (0.38–0.78)	<0.001	0.55 (0.39–0.77)	<0.001
Quartile 1	1 [Reference]		1 [Reference]		1 [Reference]	
Quartile 2	0.54 (0.38–0.77)	<0.001	0.68 (0.48–0.96)	0.028	0.67 (0.47–0.95)	0.024
Quartile 3	0.38 (0.26–0.55)	<0.001	0.55 (0.37–0.81)	0.002	0.57 (0.39–0.84)	0.004
Quartile 4	0.37 (0.25–0.56)	<0.001	0.55 (0.35–0.88)	0.012	0.54 (0.34–0.86)	0.009
*p* for trend	0.69 (0.60–0.79)	<0.001	0.80 (0.69–0.93)	<0.001	0.80 (0.69–0.93)	0.003
CVD mortality						
ln ALI	0.33 (0.23–0.46)	<0.001	0.56 (0.40–0.80)	0.002	0.56 (0.38–0.82)	0.003
Quartile 1	1 [Reference]		1 [Reference]		1 [Reference]	
Quartile 2	0.51 (0.32–0.81)	0.004	0.67 (0.42–1.09)	0.110	0.65 (0.39–1.08)	0.094
Quartile 3	0.31 (0.18–0.53)	<0.001	0.56 (0.32–0.98)	0.044	0.57 (0.32–1.02)	0.059
Quartile 4	0.25 (0.14–0.46)	<0.001	0.48 (0.25–0.93)	0.030	0.47 (0.23–0.92)	0.029
*p* for trend	0.60 (0.50–0.73)	<0.001	0.77 (0.63–0.94)	0.010	0.77 (0.62–0.95)	0.013

^a^Model 1: No adjustment for covariates; ^b^Model 2: Adjusted for age, sex, race, PIR and education level; ^c^Model 3: Adjusted for age, sex, race, PIR, education level, smoking, alcohol use, waist, AST, ALT, hypertension, hyperlipidemia, CVD, DM, and cancer.

The results from Model 3 shed light on a pronounced inverse relationship: as the ALI escalates among individuals with OA, the probability of encountering all-cause mortality undergoes a substantial decline. To illustrate, when compared to the reference cohort (Q1), the risk profiles for the subsequent Q2, Q3, and Q4 manifested as markedly reduced hazard ratios (HRs) of 0.67 (95% CI: 0.47*–*0.95), 0.57 (95% CI: 0.39*–*0.84), and 0.54 (95% CI: 0.34*–*0.86), respectively. This gradient of risk reduction is statistically significant, as evidenced by a *p* trend of 0.003. A similar pattern emerges when examining the risk of CVD mortality. Relative to the reference group, the HRs for the Q2, Q3, and Q4 groups were 0.65 (95% CI: 0.39*–*1.08), 0.57 (95% CI: 0.32*–*1.02), and 0.47 (95% CI: 0.23*–*0.92), respectively (*p* trend = 0.013).

### Non-linear relationships

**Figure 4:**
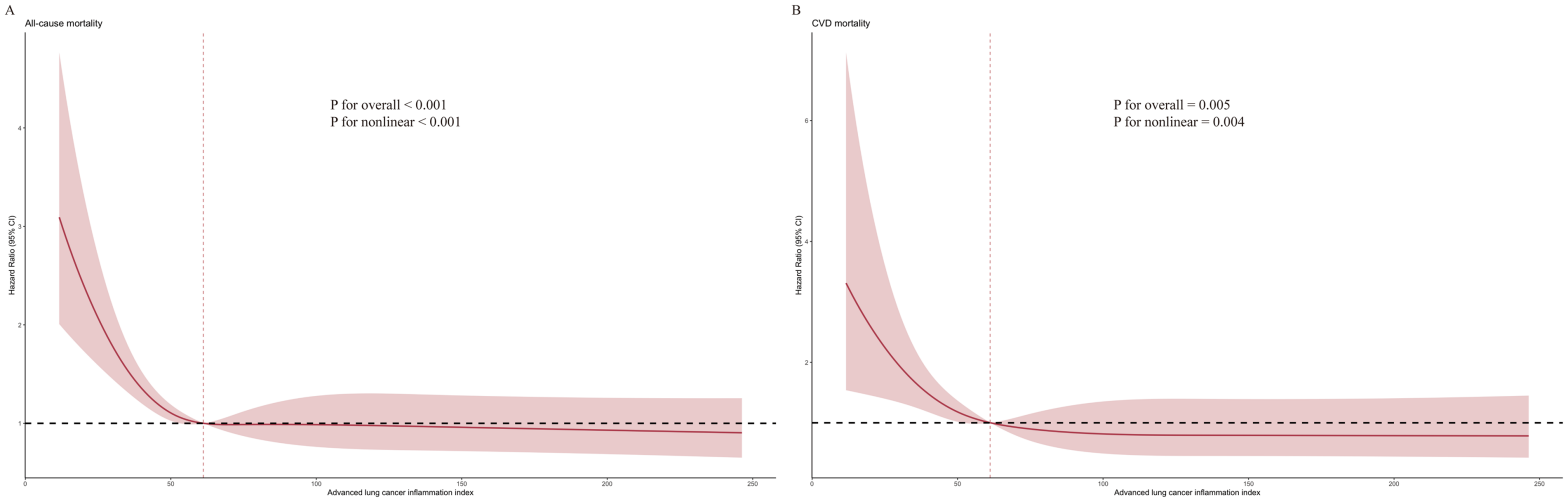
Relationship between ALI and **(A)** all-cause, and **(B)** CVD mortality in patients with OA.

Through the application of RCS analysis within Model 3, we discerned an L-shaped, non-linear association between ALI levels and the risk of all-cause mortality for patients with OA, underscored by a *p*-value of < 0.001, indicative of high statistical significance. A comparable pattern was noted for CVD mortality, achieving significance at *p* = 0.004. The inflection point for both mortality types was determined to be 61.18. For a comprehensive view, the detailed results are illustrated in Figure 4.

### Subgroup analyses

**Figure 5:**
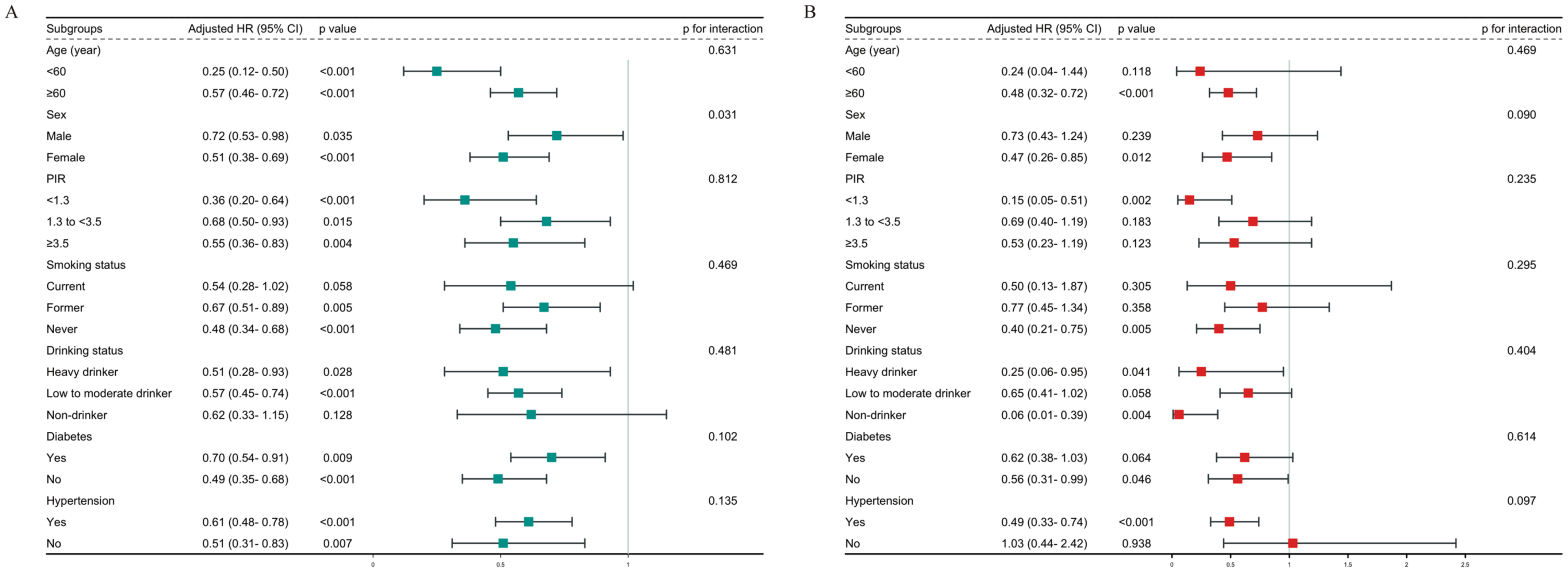
Subgroup analysis of the relationship between ALI and **(A)** all-cause mortality; **(B)** CVD mortality.

We analyzed subgroups of OA patients based on factors like gender, age, PIR, smoking, drinking, diabetes, and hypertension (Figure 5). Our analysis revealed a substantial gender-ALI interaction effect on all-cause mortality, as indicated by an interaction *p*-value of 0.031. Particularly noteworthy was the observation that the inverse relationship between elevated ALI levels and all-cause mortality risk was more pronounced among female participants compared to their male counterparts. However, the analysis of ALI in relation to CVD mortality did not find any significant interactions among the different subgroups.

## Discussion

Drawing upon data from the NHANES database, this study is among the first to explore the link between the ALI and mortality outcomes, including all-cause and CVD mortality, in individuals with OA. Upon conducting a multivariate-adjusted Cox regression analysis, we observed a marked diminishment in the hazard of both all-cause and CVD mortality corresponding to increased ALI levels within our patient cohort. Utilizing RCS analysis, we further illuminated an L-shaped, non-linear correlation between ALI and mortality outcomes, identifying a pivotal inflection point at 61.18. Subsequent subgroup analyses highlighted a significant gender interaction, where females demonstrated a stronger link between ALI and all-cause mortality. Importantly, ALI demonstrated superior predictive capability compared to other commonly used nutritional and inflammatory indicators.

OA constitutes a prominent global health problem, associated with severe disability and functional restrictions (1). Despite its traditional classification as a degenerative condition, recent investigative research has progressively underscored the pivotal role of inflammatory processes in both the initiation and progression of OA. This disorder is marked by persistent low-grade inflammation, which plays a role in synovitis, microstructural alterations in joints, and degradation of cartilage. The inflammatory mechanisms implicated in OA encompass both localized and systemic responses. Synovial tissue exhibits mild inflammation and secretes pro-inflammatory cytokines, such as interleukin-1 (IL-1) and tumor necrosis factor-alpha (TNF-*α*). These cytokines accelerate cartilage degradation and induce structural changes in bone (7, 24–26). Additionally, systemic inflammation, often linked to aging, Overweight, and systemic metabolic disorder, may accelerate the OA progressing (27, 28). As a result, elevated inflammation levels are associated with worse outcomes, aligning with our research findings. Nevertheless, earlier investigations have often concentrated on specific inflammatory markers, potentially missing a broader perspective on a patient’s inflammatory condition.

Besides inflammation, the role of nutritional status has become increasingly relevant in OA research. An expanding body of literature indicates that nutrition is crucial in both the initiation and trajectory of OA (29–32). Specifically, decreased serum albumin levels have been found to be link to an elevated susceptibility to OA (32), while a greater BMI correlates with worse OA outcomes (33, 34). Moreover, there is a complex interplay between malnutrition and inflammation (35), highlighting the importance of evaluating both elements to more precisely forecast the prognosis for OA patients.

Recent studies have increasingly focused on the prognostic value of various inflammatory and nutritional markers in OA patients. For instance, the Dietary Inflammatory Index (DII), which assesses the inflammatory potential of an individual’s diet, has been linked to all-cause mortality in OA patients. A study utilizing NHANES data from 2003 to 2018 found that higher DII scores were associated with increased mortality risk, particularly among older adults and those with comorbidities such as hypertension and cardiovascular disease (18).

Similarly, composite inflammatory markers like the Systemic Immune-Inflammation Index (SII) have demonstrated predictive capabilities for mortality outcomes. Research indicates that elevated SII levels correlate with higher all-cause and cardiovascular mortality in OA patients, suggesting that systemic inflammation plays a critical role in disease progression and outcomes (17).

In comparison, the ALI, calculated as BMI multiplied by albumin and divided by the NLR, provides a comprehensive measure of both nutritional and inflammatory status (11). Initially developed for prognostic use in lung cancer, ALI has since been applied to various inflammation-related conditions, including gastrointestinal cancers, stroke, hypertension, and diabetes (36–40). These studies consistently support the protective role of ALI, which aligns with the findings of our research.

Three principal mechanisms may shed light on the bond between ALI and the reduced mortality documented in OA cases. Firstly, the NLR is significantly linked to chronic inflammation. Changes in the NLR serve as a marker for the severity of chronic inflammation, which is essential for predicting the outcomes of inflammatory diseases (41). In OA, inflammatory cytokines such as TNF-*α*, secreted by neutrophils, engage B and T lymphocytes, perpetuating the cycle of inflammation (42). This ongoing inflammation can result in endothelial dysfunction and enhance atherosclerosis, thus increasing the likelihood of CVD mortality (43, 44). Secondly, malnutrition associated with OA involves a decrease in protein levels, potentially caused by heightened protein degradation due to inflammatory cytokines and muscle wasting from functional decline (45). Hypalbuminemia, a marker of malnutrition, is associated with an elevated risk of mortality among patients with OA (46). Thirdly, a higher BMI is associated with poorer outcomes in OA. Research indicates that an elevated BMI considerably raises the risk for knee and hand OA (33), and obesity is identified as a significant risk factor for the progression of OA (34). Sustaining a healthy BMI may serve as a protective element for individuals with OA, highlighting enhanced nutritional status and physical fitness (47). On the other hand, a very low BMI could signify malnutrition and more advanced disease, potentially resulting in a worse prognosis (48).

The subgroup analysis uncovered a significant gender-ALI interaction, as evidenced by a *p*-value of 0.031. Within the female cohort, the relationship between higher ALI levels and lower all-cause mortality was more distinct compared to the male cohort. This gender-based difference may be attributed to various factors, including the elevated occurrence and intensity of OA-related clinical symptoms in females (49), and the role of estrogen in OA pathogenesis. Emerging evidence from studies suggests that estrogen’s dual functionality in dampening inflammation and fine-tuning immune reactions could be a pivotal factor in elucidating the connection between the ALI and mortality outcomes among women afflicted with OA (50, 51). Gender-related differences in body composition, particularly fat distribution and muscle mass, are also important, as they directly affect ALI components like BMI and serum albumin (52). Additionally, psychosocial factors, such as differences in pain perception and coping mechanisms, may further explain the gender disparity in the bond between ALI and mortality susceptibility (53).

These findings have important implications for managing OA patients in clinical practice. First, the identification of 61.18 as an ALI threshold provides clinicians with a practical tool for risk assessment. Patients with ALI values below this threshold may require closer supervision and potentially additional treatment. Second, the results indicate that ALI possesses superior predictive capacity compared to other nutritional-inflammatory scores, suggesting that the implementation of ALI could be a cost-effective approach for risk profiling in individuals with OA. Furthermore, these findings imply that monitoring ALI over time may help identify higher-risk patients who would benefit from both nutritional interventions and anti-inflammatory measures. Additionally, the observed gender differences suggest that female patients with OA may derive the greatest benefit from ALI-based risk estimation.

To enhance clinical applicability, we recommend incorporating ALI assessment into routine health screenings for OA patients, particularly during initial diagnosis or annual evaluations. Patients with low ALI values may benefit from more frequent follow-up, referral to nutrition or rheumatology specialists, and early implementation of lifestyle or pharmacologic interventions. Such personalized strategies, guided by ALI-based risk stratification, may support more targeted and cost-effective management of OA in diverse clinical settings.

In our study, the selection of the ALI threshold at 61.18 was data-driven and derived from RCS analysis, which modeled the non-linear relationship between ALI and mortality risk. The inflection point of 61.18 represents the value at which the hazard ratio curve begins to flatten, indicating a diminishing marginal benefit of higher ALI values beyond this point. This suggests that patients with ALI below 61.18 are at significantly elevated risk and may benefit most from clinical attention. While this threshold enhances risk stratification in OA populations, further prospective studies are warranted to validate its generalizability and to refine its use across different demographic and clinical subgroups. Nonetheless, the RCS-derived cut-off adds a meaningful and statistically supported reference for guiding clinical decisions in OA management.

This study has several key strengths. The analytical techniques we employed were statistically robust. In order to enhance the validity of our findings. we applied Cox regression analysis and adjusted for multiple potential confounding factors. Additionally, the use of RCS analysis allowed for the detection of nonlinear relationships and the precise determination of the ALI level. Lastly, we compared ALI with other inflammatory markers and identified its advantages in assessing nutritional inflammatory status.

Interpreting the findings of our study necessitates awareness of certain limitations. Primarily, the cross-sectional methodology employed in this research suggests that the association between ALI and the risk of mortality ought to be regarded as correlational, not causal. Additionally, the use of NHANES, an observational database, introduces the possibility of residual confounding, despite our adjustment for a comprehensive range of covariates. Some unmeasured or unknown factors—such as medication use, dietary patterns, or disease severity—may have influenced both ALI levels and mortality risk. Furthermore, the components used to calculate ALI (BMI, albumin, and neutrophil count) are subject to potential measurement variability. BMI may not fully reflect body composition or nutritional status, albumin levels can be affected by acute illness or fluid status, and neutrophil counts may fluctuate in response to transient infections or stress. These factors may introduce non-differential misclassification, potentially attenuating the observed associations. Lastly, as the NHANES cohort is representative of the U.S. population, generalizability to other ethnic or geographic populations may be limited.

Additionally, although NHANES offers broad data, it does not include detailed information regarding OA severity, joint distribution, treatment options, and other variables that could affect mortality risk. Lastly, while we measured ALI at baseline, we did not have the capacity to evaluate how changes in ALI over time might influence mortality risk.

## Conclusion

Our comprehensive analysis reveals that an increase in ALI is associated with a significant reduction in the incidence of both all-cause and cardiovascular mortality among patients diagnosed with OA. Notably, ALI demonstrated robust predictive performance, particularly among female patients, suggesting potential sex-based differences in inflammatory and nutritional pathways. These findings underscore the clinical relevance of ALI as a readily available, cost-effective, and easily interpretable biomarker that could be integrated into routine risk assessment and management strategies for OA. Incorporating ALI into clinical evaluation may help identify high-risk individuals who could benefit from targeted interventions aimed at improving nutritional and inflammatory status. Further research is warranted to elucidate the underlying mechanisms and validate the prognostic utility of ALI in diverse OA populations, thereby enhancing its role in advancing personalized medicine.

## Data Availability

The original contributions presented in the study are included in the article/Supplementary material, further inquiries can be directed to the corresponding author.

## Glossary

OA: Osteoarthritis, a degenerative joint disorder characterized by cartilage loss and synovial inflammation
ALI: Advanced Lung Cancer Inflammation Index, a composite metric derived from serum albumin, lymphocyte count, and neutrophil count, used to assess systemic inflammation and prognostic risk
CVD: Cardiovascular Disease, includes conditions such as stroke, angina, myocardial infarction, coronary heart disease, and heart failure
NHANES: The National Health and Nutrition Examination Survey, is a U.S.-based study that offers comprehensive cross-sectional data on the health and nutritional status of the population
RCS: Restricted Cubic Spline, a statistical modeling technique used to evaluate non-linear relationships between variables while controlling for confounding
ROC: Receiver Operating Characteristic curve, a graphical tool to assess diagnostic test accuracy by plotting sensitivity vs. 1-specificity
CRP: C-reactive protein, an acute-phase protein elevated during inflammation, used as a biomarker for systemic immune activation
ESR: Erythrocyte Sedimentation Rate, a laboratory test reflecting inflammatory activity through the rate of red blood cell sedimentation
BMI: Body Mass Index, calculated as weight (kg)/height (m^2^), a proxy for adiposity and metabolic risk
NLR: Neutrophil-to-Lymphocyte Ratio, a blood-based marker of immune dysregulation, elevated in inflammatory and infectious states
PIR: Income-Poverty Ratio, a socioeconomic indicator defined as household income divided by the federal poverty threshold, used to stratify economic status
DM: Diabetes Mellitus, a sustained metabolic disorder exhibiting hyperglycemia and insulin opposition, a key risk factor for CVD
ALT/AST: Alanine Aminotransferase/Aspartate Aminotransferase, liver enzymes used to evaluate hepatic function and detect hepatocellular injury
OR: Odds Ratio, a measure of association between exposures and outcomes in case–control or cross-sectional studies
HR: Hazard Ratio, a ratio of event rates comparing exposed vs. unexposed groups over time, used in survival analysis to quantify mortality risk
