# Supplementary material for: Advanced lung cancer inflammation index and its predictive value for all-cause and cardiovascular mortality among osteoarthritis patients: a NHANES-based study from 2001 to 2018
Source: Front Med (Lausanne). 2025 May 27;12:1593374. doi: 10.3389/fmed.2025.1593374 (PMC12150347; doi:10.3389/fmed.2025.1593374)
Supplement: Supplementary file 1 [file Data_Sheet_1.docx]

**Table S1. Calculation of various nutritional/inflammatory indicators.**

| **Indicators** | **Definition or calculation formula** |
| --- | --- |
| **ALI** | BMI (kg/m^2^) × albumin (g/dl) / NLR |
| **NLR** | neutrophil count (×10^9^) / lymphocyte count (×10^9^) |
| **SII** | platelet count (×10^9^) × neutrophil count (×10^9^) / lymphocyte count (×10^9^) |
| **SIRI** | neutrophil count (×10^9^) × monocyte count (×10^9^) / lymphocyte count (×10^9^) |
| **PIV** | neutrophil count (×10^9^) × platelet count (×10^9^) × monocyte count (×10^9^) /lymphocyte count (×10^9^) |
| **PNI** | albumin (g/L) + 5×lymphocyte count (×10^9^) |
| **NPAR** | Neutrophil percentage (in total WBC count) (%) × 100/Albumin (g/dL) |
| **TCBI** | Triglycerides (mg/dL) × Total Cholesterol (mg/dL) × Body Weight (kg)/1,000 |
| **GNRI** | 1.489×albumin (g/L) + (41.7× current weight/ ideal body weight (IBW) |
| **CONUT score** |  |
| Serum albumin (g/L) | albumin score |
| ≥ 35 | 0 |
| 30 to < 35 | 2 |
| 25 to < 30 | 4 |
| < 25 | 6 |
| Total lymphocytes (×10^9^/L) | Total-lymphocyte score |
| > 1.6 | 0 |
| 1.2 to 1.6 | 1 |
| 0.8 to <1.2 | 2 |
| < 0.8 | 3 |
| Total cholesterol (mg/dL) | Total-cholesterol score |
| > 180 | 0 |
| 140-180 | 1 |
| 100 to < 140 | 2 |
| < 100 | 3 |
| CONUT score (total) | albumin score+ TLC score+ Total-cholesterol score |

Abbreviation: ALI, advanced lung cancer inflammation index; NLR, neutrophil to lymphocyte ratio; SII, systemic immune-inflammation index; SIRI, systemic inflammation response index; PIV, pan-immune-inflammation value; PNI, prognostic nutritional index; NPAR, Neutrophil Percentage-to-Albumin Ratio; TCBI, Triglycerides (TG) × Total Cholesterol (TC) × Body Weight (BW) Index; GNRI, geriatric nutritional risk index; COUNT score, controlling nutritional status score.
